# Towards Accurate, Cost-Effective, Ultra-Low-Power and Non-Invasive Respiration Monitoring: A Reusable Wireless Wearable Sensor for an Off-the-Shelf KN95 Mask †

**DOI:** 10.3390/s21206698

**Published:** 2021-10-09

**Authors:** Yu Xu, Qi Li, Zhenzhou Tang, Jun Liu, Bingjin Xiang

**Affiliations:** College of Computer Science and Artificial Intelligence, Wenzhou University, Wenzhou 325035, China; 194511981416@stu.wzu.edu.cn (Q.L.); tzz@wzu.edu.cn (Z.T.); junliu@wzu.edu.cn (J.L.); xbj@hdu.edu.cn (B.X.)

**Keywords:** airflow temperature, non-invasive respiration monitoring, wireless wearable sensor

## Abstract

Respiratory rate is a critical vital sign that indicates health condition, sleep quality, and exercise intensity. This paper presents a non-invasive, ultra-low-power, and cost-effective wireless wearable sensor, which is installed on an off-the-shelf KN95 mask to facilitate respiration monitoring. The sensing principle is based on the periodic airflow temperature variations caused by exhaled hot air and inhaled cool air in respiratory cycles. By measuring the periodic temperature variations at the exhalation valve of mask, the respiratory parameters can be accurately and reliably detected, regardless of body movements and breathing pathways through nose or mouth. Specifically, we propose a voltage divider with controllable resistors and corresponding selection criteria to improve the sensitivity of temperature measurement, a peak detection algorithm with spline interpolation to increase sampling period without reducing the detection accuracy, and effective low-power optimization measures to prolong the battery life. The experimental results have demonstrated the effectiveness of the proposed sensor, showing a small mean absolute error (MAE) of 0.449 bpm and a very low power consumption of 131.4 μW. As a high accuracy, low cost, low power, and reusable miniature wearing device for convenient respiration monitoring in daily life, the proposed sensor holds promise in real-world feasibility.

## 1. Introduction

Respiratory rate is an important vital sign reflecting the state of human body. Abnormal respiratory rate is an early diagnostic indicator for many diseases related to lung and heart. For example, a respiratory rate of ≥30 bpm is an indicator for severe clinical type of COVID-19 [1], a respiratory rate of >27 bpm is a better predictor of cardiopulmonary arrest within 72 h than heart rate, as well as systolic blood pressure [2], and a respiratory rate of ≤8 bpm is associated with 18.1 times the odds of death within one day compared to normal respiratory rate [3]. Respiratory rate is also essential to classify sleep stages [4], as well as to detect obstructive sleep apnea, which has a certain chance of sudden death and an estimated prevalence of 3–7% in men and 2–5% in women [5]. In addition, respiratory rate reflects exercise intensity more quickly than heart rate, hence can be used to actively control exercise intensity [6]. Therefore, continuous and accurate monitoring of respiratory rate is very crucial for early diagnosis of disease, and monitoring of sleep quality or exercise intensity. However, respiratory rate is often frequently omitted, inaccurately measured and not recorded [7].

In hospitals, visual observation [8], thoracic impedance pneumography [9] and Capnography [10] are the primary clinical tools for respiration monitoring. However, due to cost and the need for professional operation, these methods are generally only used during anesthesia or intensive care. Hence, even in hospitals, the respiratory rate is often the most poorly recorded vital sign [11], not to mention the monitoring in daily life. With the rapid development of Internet of Things (IoT) technology in recent years, there has been considerable attention from researchers to provide ubiquitous low-cost respiration monitoring approaches, which can be classified into three categories: ECG/PPG based indirect approaches, chest or abdomen movement based approaches, and respiratory airflow based approaches.

ECG/PPG based indirect approaches extract respiration signals from the electrocardiogram (ECG) [12] or photoplethysmogram (PPG) [13] signals, which exhibit amplitude and frequency oscillations reflecting respiratory rate. Due to the professional installation of electrodes, expensive monitors, and restricted body movements, the application of these approaches in daily life is greatly limited.

Chest or abdomen movement based approaches measure respiratory rate by sensing the vibrations [14], pressure changes [15,16,17,18,19], displacement changes [20,21,22,23,24,25,26,27,28,29,30,31,32,33], or bioelectrical signals [34,35] caused by chest or abdomen movements during respirations. These approaches can provide respiration monitoring at fixed locations [14,15,16,17,21,22,23,24,25,26,27,28,29,30,31,32], such as specific beds, mattresses, and rooms, or in wearable ways [18,19,20,30,31,32,33,34,35]. However, due to the coupling of respiratory motion and other body movements, reducing the influence of artefacts caused by other body movements is also challenging. In addition, obstructive apnoea may go undetected because the chest wall continues to move as the patient attempts to breathe [36].

Respiratory airflow based approaches measure respiratory rate by sensing the changes of sound, temperature, or humidity caused by inhalation of fresh air and exhalation of warm humid air. The changes of temperature around nose and mouth can be detected with FIR cameres [37,38]. The envelop of breath sound is also a respiratory sign that can be captured by microphone [39,40]. The acoustic radar [41] utilizes a speaker transmitting inaudible ultrasound waves and a microphone receiving back scattered echoes to detect periodic Doppler frequency shift caused by exhaled airflow. However, the thermal images and sounds may be considered obtrusive in terms of personal privacy. To achieve the goal of mobile monitoring in daily life, a variety of wearable solutions have been proposed. These solutions take advantage of piezoelectric membrane sensors [42], negative temperature coefficient (NTC) resistors [43], hot-film resistors [44], micromachined planar capacitors [45], and alveolus-inspired membrane sensors [46] to sense pressure [42], temperature [43,44], humidity [45], and nitrogen dioxide [46] variations caused by exhaled airflow. These sensors, which are attached underneath the nose, are insensitive to body movement, but cannot detect respiration while airflow is exhaled through the mouth. This paper is an extended version of [47], where we introduced our preliminary approach to detect respiration. In this paper, we propose a miniaturized, ultra-low-power, and reusable sensor attaching to an off-the-shelf KN95 mask to implement wireless, non-invasive, real-time, ambulatory, and accurate monitoring of respiration in daily life. The sensor leverages five NTC series resistors installed near vent holes of KN95 mask to sense the temperature of respiratory airflow, and a Bluetooth low energy (BLE) system-on-chip (SoC) to extract the respiratory rate, inspiratory time, expiratory time of each respiratory cycle, and the occurrence time, duration of each apnea by analyzing the temperature variations of respiratory airflow. The sensor can achieve accurate detection of respiration at low sampling frequency and low power consumption. In addition, the system based on the KN95 mask has better wearing comfort and detection convenience.

The rest of paper is organized as follows. The system design methodology and detailed design are illustrated in Section 2. We provide the experimental results and discussion in Section 3 and Section 4. Finally, the work is concluded in Section 5.

## 2. Materials and Methods

### 2.1. Sensing Principle

**Figure 1:**
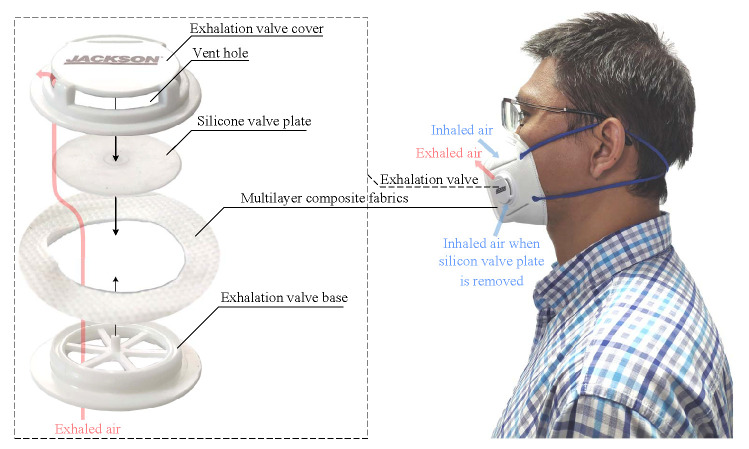
The structure of a KN95 mask with exhalation valve.

Figure 1 shows the structure of a KN95 mask with exhalation valve (Kimberly-Clark 63207V). The exhalation valve, which are tightly fastened onto the multilayer composite fabrics, consists of a plastic valve base, a silicone valve plate and a plastic valve cover. When inhaling with the mask, the intercostal muscles contract, increasing the size of the thoracic cavity and creating a negative pressure. The negative pressure makes the valve plate seal with the base, so that the fresh air can only be drawn into the lung through the multilayer composite fabrics. During exhaling, the inspiratory muscles relax and the lung contracts, creating a positive pressure. The positive pressure blows open the valve plate and exhausts the hot air quickly. As the body temperature is higher than the ambient temperature, the temperature at the vent holes of exhalation valve increases during exhalation and decreases during inhalation. Since the mouth and nose are covered by the fabrics of mask, most of the exhaled air comes out through the exhalation valve. Therefore, respiration can be detected by sensing the airflow temperature at the vent holes, regardless of breathing pathways through nose or mouth.

**Figure 2:**
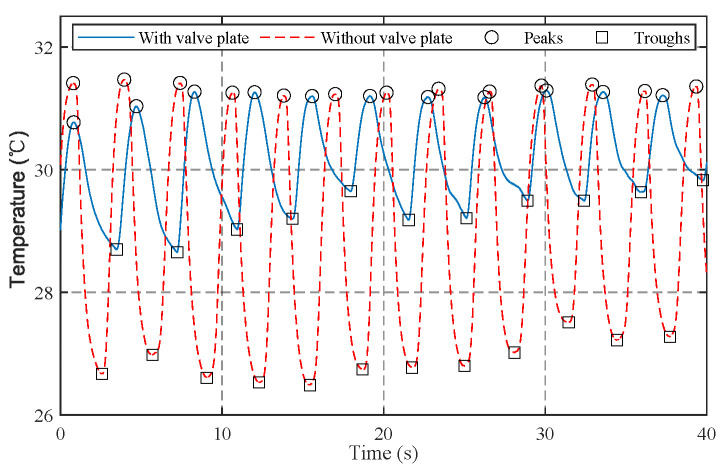
The measured temperatures at the vent hole of valve.

To enhance comfort, the silicone valve plate can be removed. This will disable the protection function of mask, but the air can be inhaled more smoothly through the exhalation valve instead of the fabrics, making the mask more suitable for respiration monitoring in exercises. In addition, compared with the static air, the inhaled air can decrease the temperature at the vent holes more effectively. As a result, the temperature and humidity change more significantly. Figure 2 shows the temperature signals at the vent hole of exhalation valve measured with a sample frequency of 100 Hz and a lowpass frequency of 1 Hz. The average temperature variations (peak minus trough) are 1.89 ${}^{\circ }$C with valve plate and 4.44 ${}^{\circ }$C without valve plate. In both situations the peaks and troughs can be accurately detected by the peak detection algorithm [43].

### 2.2. System Architecture

**Figure 3:**
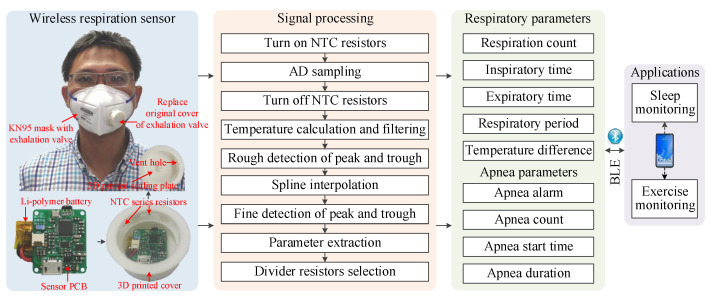
The system architecture of wireless respiration sensor.

As shown in Figure 3, the sensor consists of a sensor printed circuit board (PCB), five NTC series resistors, a Li-polymer battery, a 3D printed cover, and a 3D printed sealing plate. The five NTC series resistors are, respectively, installed near the five vent holes of the 3D printed cover, and connects to the sensor PCB installed in the 3D printed cover with a 1.27 mm 2-pin connector. To prevent exhalation airflow from escaping, a 3D printed sealing plate is mounted on the cover. So that the vent holes become the main pathway of exhaled air. The mounting interface of the 3D printed cover is carefully designed according to the original cover of the exhalation valve, hence it can be easily fastened to and disassembled from the exhalation valve base without any additional assembly unit, making the sensor reusable. In addition, the sensor can be adapted to other off-the-shelf masks by 3D printing matched covers. The sensor leverages a signal processing pipeline running on the BLE SoC of sensor PCB to extract respiratory parameters and apnea parameters from the resistance variations induced by respiratory airflows in realtime. The signal processing pipeline uses a set of controllable divider resistors, as well as the corresponding selection criteria to improve the measuring sensitivity of temperature. A peak detection algorithm has been developed to extract parameters of every respiratory cycle and apnea event. Moreover, a spline interpolation algorithm is proposed to reduce sampling frequency while retaining measurement accuracy, resulting in a significant reduction in power consumption. The respiratory parameters and apnea parameters are finally transmitted to smartphone via BLE 4.0 interface for applications, such as sleep monitoring and exercise monitoring.

### 2.3. Hardware Design

**Figure 4:**
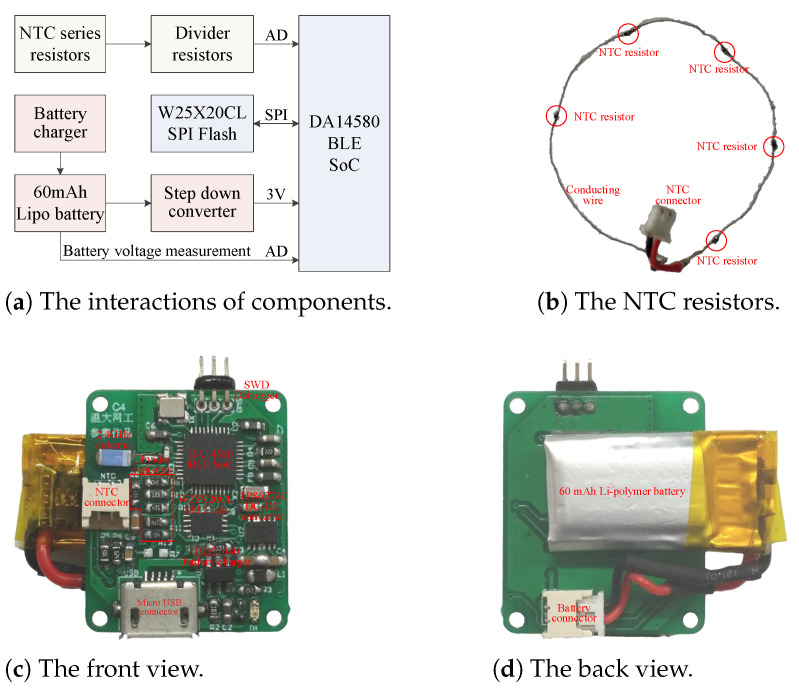
The block diagram and realization of sensor hardware.

Accuracy, cost, power consumption, weight, as well as size, are all factors to be considered in hardware design. Figure 4 illustrates the block diagram and realization of sensor hardware. Five NTC resistors in series are connected to sensor PCB with a 1.25 mm connector. The sensor PCB consists of a battery charger, a SPI Flash, a step down voltage converter, divider resistors, a BLE SoC and corresponding support circuits. The total 38 components are all off-the-shelf and low in price. In addition, due to the small size and high integration of components, the sensor PCB measures only 24 × 20 mm${}^{2}$, which is small enough to be installed into the exhalation valve.

For long battery life, low battery cost and light battery weight, the sensor should minimize its power consumption. This aim is achieved by using low power and high efficiency components, such as the DA14580 low power BLE SoC with less than 4.9 mA radio current and 0.6 μA sleeping current, the TPS62740 ultra-low-power step-down buck DC–DC converter with up to 90% efficiency at 10 μA output current and a ultra low quiescent current of 0.36 μA, and the W25X20CL SPI Flash memory with 1 mA active current and 1 μA power-down current. In addition, the battery can be recharged by the BQ21040 battery charger via the micro USB connector.

**Figure 5:**
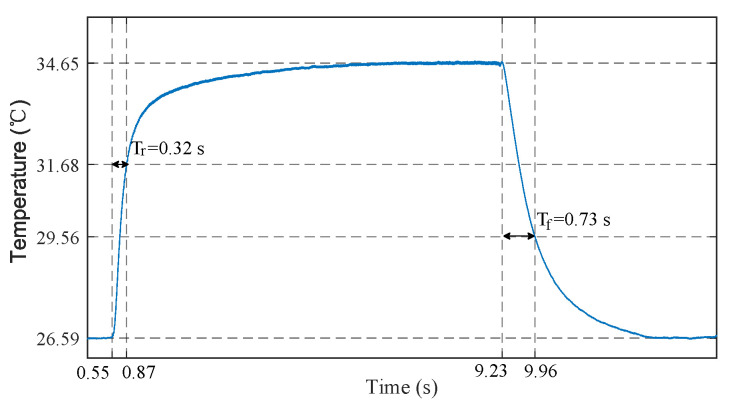
The step response of NTC temperature sensor, during which the sensor was heated with body temperature (finger) and cooled with ambient air.

**Figure 6:**
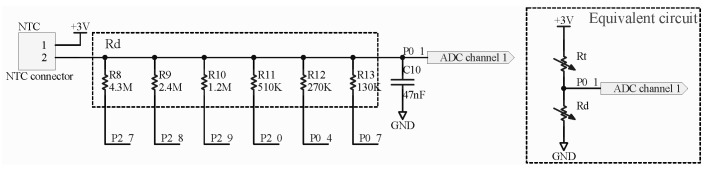
The voltage divider with controllable resistors.

To accurately detect light breath during sleep and fast breath during exercise, the sensor should be provided with both fast response time (small time constant) and large measurement gain. The time constant of sensor is defined as the time required for the sensor output to reach to 63.2% of its total step change. Smaller mass, smaller surface area, and potting material with higher thermal conductivity help to reduce the time constant of sensor. Hence we select the Murata NCP15WL473J03RC NTC resistor considering its negligible mass, small size of 1 × 0.5 × 0.5 mm${}^{3}$, ceramic potting, and large B-constant. To measure its time constant, we conducted a step response experiment, in which the NTC resistor was heated with body temperature (finger) and cooled with ambient air. As shown in Figure 5, the measured time constants of heating and cooling are 0.32 s and 0.73 s, respectively. Generally, the resistance of NTC resistor can be converted to voltage signal by a Wheatstone bridge and subsequently amplified by a differential amplifier to increase the measurement gain [44]. However, these components also introduce additional power consumption. Resistance voltage divider is another low power method, but the constant divider resistor cannot maintain a high measurement gain over a wide temperature range. In order to solve the contradiction between measurement gain and power consumption, we leverage a set of controllable divider resistors and corresponding selection criteria to convert the resistance of NTC resistors to voltage signals. For a resistance voltage divider shown in Figure 6, the output voltage can be expressed as

(1) $$V=\frac{R_{d}}{R_{d}+R_{t}}V_{p}=\frac{R_{d}}{R_{d}+R_{0}e^{B(1/T-1/T_{0})}}V_{p},$$

where $R_{d}$ and $R_{t}$ are the resistances of divider resistor and NTC resistor, respectively, $V_{p}=3$ V is the constant supply voltage, $R_{0}=5\times 47\;k$Ω is the resistance value of five NTC series resistors in 25 ${}^{\circ }$C, B=4485 is the B-constant of NTC resistor, *T* is the measured absolute temperature at the vent holes of exhalation value and $T_{0}=298.15$ K is the absolute temperatures of 25 ${}^{\circ }$C. The output voltage is then sampled by the 10-bit integrated ADC of BLE SoC. The ADC sampling result *N* is proportional to the output voltage

(2) $$N=\frac{2^{10}V}{V_{\mathrm{ref}}}=\frac{1024V_{p}R_{d}}{\left[R_{d}+R_{0}e^{B(1/T-1/T_{0})}\right]V_{\mathrm{ref}}},$$

where $V_{\mathrm{ref}}$ is the reference voltage of ADC. By derivation of (2) respect to *T*, we yield

(3) $$\begin{array}{c} \frac{dN}{dT}=\frac{1024BV_{P}R_{d}R_{0}e^{B(1/T-1/T_{0})}}{T^{2}V_{\mathrm{ref}}{\left[R_{d}+R_{0}e^{B(1/T-1/T_{0})}\right]}^{2}}\\ =\frac{1024BV_{P}}{T^{2}V_{\mathrm{ref}}\left(2+R_{d}/R_{t}+R_{t}/R_{d}\right)}. \end{array}$$

**Figure 7:**
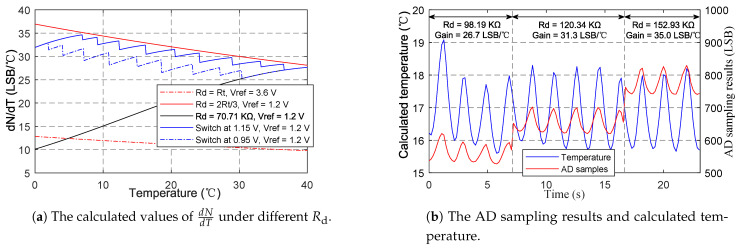
The verification of voltage divider with controllable resistors.

As can be seen from (3), the gradient of sampling result respect to temperature $\frac{dN}{dT}$ is determined by the B-constant *B*, the reference voltage $V_{\mathrm{ref}}$, and the resistance of divider resistor $R_{d}$. To improve the measurement gain, larger *B* and smaller $V_{\mathrm{ref}}$ are preferred. Hence we select the NCP15WL473J03RC NTC resistor with a large B-constant of 4485, and the $V_{\mathrm{ref}}$ of 1.2 V among the $V_{\mathrm{ref}}$ of 1.2 V and 3.6 V provided by the ADC. $R_{d}$ affects $\frac{dN}{dT}$ in a complex way. $\frac{dN}{dT}$ increases monotonously with $R_{d}$ when $R_{d}<R_{t}$, decreases monotonously with $R_{d}$ when $R_{d}>R_{t}$, and achieves its maximum when $R_{d}=R_{t}$. As $R_{t}$ changes with temperature, a constant $R_{d}$ cannot remain high measurement gain over wide temperature range. Ideally, to achieve the maximal $\frac{dN}{dT}$, $R_{d}$ should always equal to $R_{t}$. However, under this condition ($R_{d}=R_{t}$), the output voltage computed from (1) is 1.5 V, exceeding the ADC input range defined by $V_{\mathrm{ref}}=1.2\;V$. For $V_{\mathrm{ref}}=1.2\;V$, the maximal $\frac{dN}{dT}$ is achieved when the output voltage of voltage divider is 1.2 V, corresponding to $R_{d}=\frac{2}{3}R_{t}$ derived from (1). Hence the voltage divider with controllable divider resistors has been adopted to replace the simple voltage divider with a constant resistance. As shown in the Figure 6, R8−R13 are, respectively, controlled by the 6 general purpose input output (GPIO) pins of BLE SoC. When a GPIO pin outputs zero, the corresponding resistor is connected to ground and changes $R_{d}$. When a GPIO pin is configured as input, the corresponding resistor is in high-impedance state and does not change $R_{d}$. Therefore, a set of resistance values can be obtained by controlling the corresponding GPIO pins. In practical application, we change $R_{d}$ according to the following strategy: switch to smaller $R_{d}$ to ensure measuring range when the output voltage exceeds 1.15 V, and switch to larger $R_{d}$ to increase measurement gain when the peak voltage is below 0.95 V over 5 respiratory cycles. The calculated values of $\frac{dN}{dT}$ under different $R_{d}$ shown in Figure 7a indicate that the proposed switching strategy can achieve high measurement gains over the entire temperature range. Figure 7b shows the effectiveness of the strategy in practical application. $R_{d}$ gradually switches to higher resistance values, and the gain ($\frac{dN}{dT}$) increases accordingly. During the switching process, although the AD sampling value changed suddenly, the calculated temperature is still not affected. A high gain of about 35.0 LSB/${}^{\circ }$C can be achieved. In contrast, the gain for constant $R_{d}$ (70.71 KΩ) at 17 ${}^{\circ }$C is only 18.9 LSB/${}^{\circ }$C, and the gain may further decrease with the decrease in temperature.

### 2.4. 3D Printed Cover Design and Sensor Assembly

**Figure 8:**
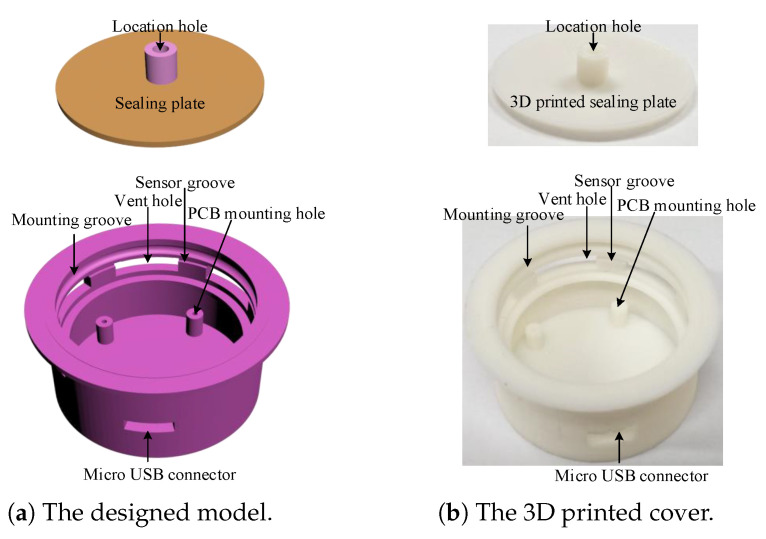
The design and realization of the exhalation valve cover.

Since KN95 masks are consumables and need to be replaced frequently, the sensor should be easy to reuse. The reusability of sensor is realized by the 3D printed exhalation valve cover shown in Figure 8. The sensor PCB with a battery attached to its back shown in Figure 4c,d is fixed on the mounting holes by four screws. The NTC series resistors shown in Figure 4b are glued to the sensor groove, so that the five resistors are just located at the five vent holes, respectively. To prevent the exhaled airflow from escaping, the sensor PCB is covered by a 3D printed sealing plate, making the five vent holes to be the main pathways of exhaled airflow. The 3D printed exhalation valve cover is designed with a mounting groove, which just matches the convex ring on the exhalation valve base. The cover and the base can be tightly fastened with the groove ring and the convex. In addition, the cover and the base can also be easily separated by force. Therefore, the sensor can be conveniently reused by replacing the original cover of the KN95 mask (Kimberly-Clark 63203V/63207V/63310V). The sensor can also adapt to other masks by redesigning matching covers.

The assembled sensor weighs 11.21 grams and measures 44 × 44 × 18 mm${}^{3}$. Compared with the original exhalation valve cover of 2.27 g, it only adds a small weight of 8.94 g, imposing negligible impact on wearing comfort and appearance of a KN95 mask. Cost is also an important consideration for large scale application. The assembled sensor costs as little as USD 3.67. Moreover, the sensor can be reused to further reduce cost.

### 2.5. Signal Processing and Algorithms

The signal processing pipeline shown in Figure 3 runs on the BLE SoC at a fixed sampling period $T_{s}$. At the beginning, the NTC resistors is turned on by setting corresponding GPIO pins of selected divider resistors to zero, and turned off immediately by configuring the GPIO pins as inputs after AD sampling to reduce power consumption. The temperature *T* can be computed by substituting the sampling result *N* into (2). The temperature is then filtered by a first order low-pass digital filter with cut-off frequency of 1Hz to reduce high-frequency noise.

(4) $$T_{f}\left(n\right)=\frac{0.1592T_{f}\left(n-1\right)+T_{s}T\left(n\right)}{0.1592+T_{s}}.$$

where $T_{f}\left(n\right)$ is the filtered temperature at the n-th sampling period. During respirations, the NTC resistors are alternately heated by exhaled airflow and cooled by ambient air, resulting in periodic temperature changes. The interval between two consecutive peaks correspond to the respiratory period. We adopt a peak detection algorithm [43] to roughly detect the peaks and troughs. The algorithm detects peaks (or troughs) by searching the local maximum (or minimum) and the subsequent lower point (or higher point) with an absolute difference larger than the detection threshold. To accommodate different respiration intensities, the detection threshold is set to 0.2 ${}^{\circ }$C for sleep monitoring and 0.4 ${}^{\circ }$C for exercise monitoring, respectively. Figure 2 demonstrates that the peaks and troughs can be accurately detected at a sampling period of 10 ms.

**Figure 9:**
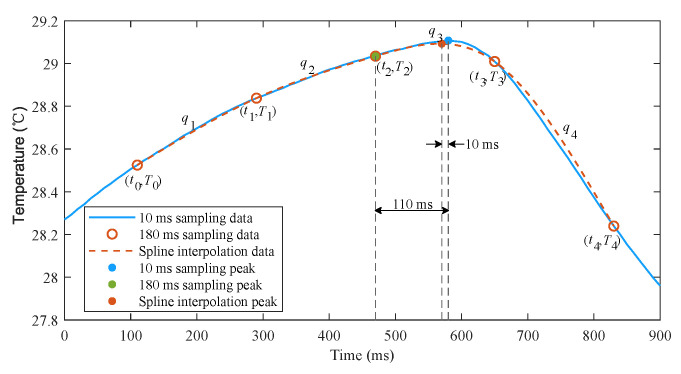
Cubic spline interpolation of temperature samples.

However, there is a trade-off between detection accuracy and power consumption. A smaller sampling period helps to improve detection accuracy while a larger sampling period reduces power consumption. In order to achieve a good trade-off between accuracy and power consumption, we implement a cubic spline interpolation algorithm to reconstruct smooth temperature curve from sparse sampling points. Figure 9 shows that the proposed interpolation algorithm can well reproduce the temperature signals using only five samples, and the detection error is dramatically reduced. The interpolation procedure is as follows.

(1) Once a peak or a trough with recorded time instant $t_{2}$ and temperature $T_{2}$ is detected by the rough detection algorithm, obtain the previous two samples recorded as ($t_{0}$, $T_{0}$), ($t_{1}$, $T_{1}$), and the subsequent two samples recorded as ($t_{3}$, $T_{3}$), ($t_{4}$, $T_{4}$);

(2) Calculate the smooth temperatures using interpolation equations $q_{1}$–$q_{4}$ at a small interval of 10 ms, where $q_{i}$ is expressed as follows

(5) $$\begin{array}{cc} q_{i}\left(x\right) & =\left(1-x\right)T_{i-1}+xT_{i}+x\left(1-x\right)\left[a_{i}\left(1-x\right)+b_{i}x\right]\\ x & =\left(t-t_{i-1}\right)/T_{s},t_{i-1}\leq t<t_{i} \end{array}.$$

$a_{i}$ and $b_{i}$ are the coefficients that satisfy the twice continuously differentiable condition and natural spline as follows

(6) $$\begin{array}{cc} {q^{{}^{\prime }}}_{i}\left(1\right) & ={q^{{}^{\prime }}}_{i+1}\left(0\right),i=1∼3\\ {q^{{}^{\prime \prime }}}_{i}\left(1\right) & ={q^{{}^{\prime \prime }}}_{i+1}\left(0\right),i=1∼3\\ {q^{{}^{\prime \prime }}}_{1}\left(0\right) & ={q^{{}^{\prime \prime }}}_{4}\left(1\right)=0 \end{array}.$$

When a trough and a subsequent peak are detected, the time stamps of previous peak and current trough can be recorded as the inspiratory time and the expiratory time, and the interval between two consecutive peaks can be recorded as respiratory period. If the inspiratory or expiratory period and the temperature change are too small, the current fake trough or peak need to be discarded, and there must be a fake peak or trough between the previous and subsequent two peaks or troughs. Then, the peak with higher temperature or the trough with lower temperature is determined as the true peak or trough. If no respiration is detected for a long time, an apnea is considered to occur. Once a new peak is redetected, the parameters of current apnea can be calculated. As will be mentioned in Section 3, the algorithm can effectively improve detection accuracy and greatly reduce power consumption.

### 2.6. Low Power Design

Low power consumption not only helps to improve battery life, but also reduces capacity, size, weight, and cost of battery. In addition to the selection of low power and high efficiency components in hardware design, the following comprehensive optimization measures have been taken to further reduce power consumption.

1. Once the signal processing pipeline has been executed, turn off non-essential components until the next sampling period. The BLE SoC goes into extended sleep mode, all the controllable divider resistors are set to high-impedance state, and the Flash memory is also powered down by instruction to reduce standby current;
2. To reduce wakeup events caused by BLE connection, the connection interval and slave latency are changed from 30 ms and 0 to 180 ms and 54;
3. Reduce the runtime of the signal processing pipeline. The level 2 and time optimizations of complier have been configured, and the codes have been optimized by merging calculation steps and using single floating point variables instead of double floating point variables. In addition, the CORDIC algorithm [48] has been implemented to accelerate the computation of natural logarithm function, so that its average computation time decreases from 774.5 μs to 66.4 μs. The above measures reduce the average runtime of signal processing pipeline from 1488.8 μs to 277.6 μs with a sampling period of 30 ms;
4. Increase the sampling period to reduce the duty cycle, and implement the cubic spline interpolation algorithm to compensate for the negative impact of large sampling period.

### 2.7. End User Application

**Figure 10:**
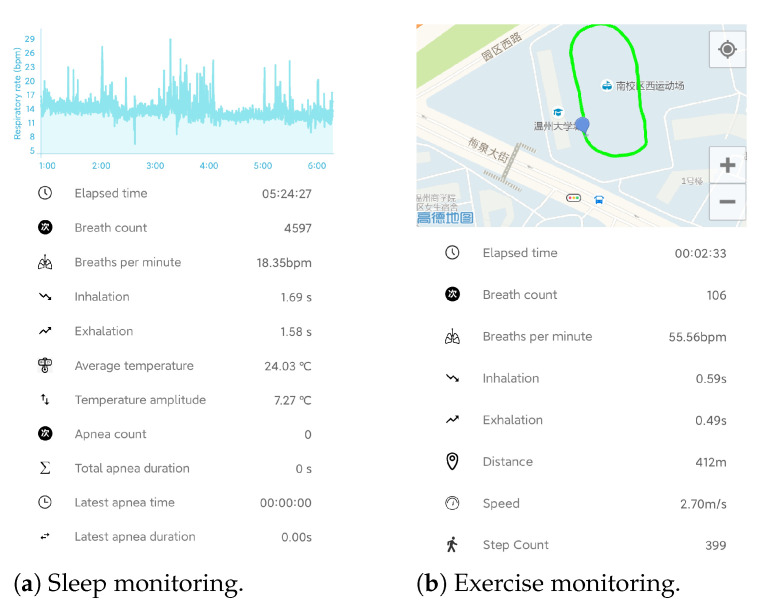
The Android APP for real time respiration monitoring.

In order to monitor respiration in real time, an Android APP used for sleep monitoring and exercise monitoring has been developed, as shown in Figure 10. The respiratory parameters, as well as apnea events in sleep and motion parameter in exercise can be displayed in real time and stored for further analysis. In sleep monitoring, the silicone valve plate can be removed to smooth the respiratory airflow and improve wearing comfort.

## 3. Results

### 3.1. Overview of Experiments

We conducted a series of experiments to verify the effectiveness, accuracy, and power consumption of our proposed sensor.

**Figure 11:**
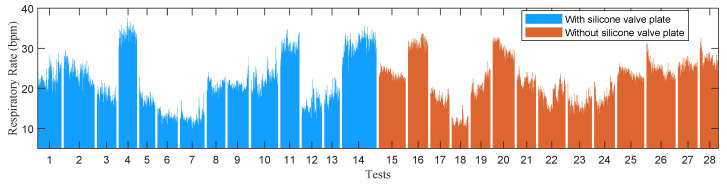
The respiratory rates of 28 tests measured by the button in the experiments of stationary mode.

**Figure 12:**
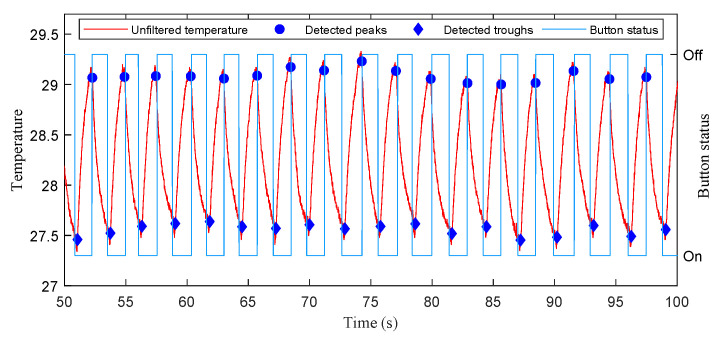
The recorded temperature and button status in a test of stationary mode.

**Figure 13:**
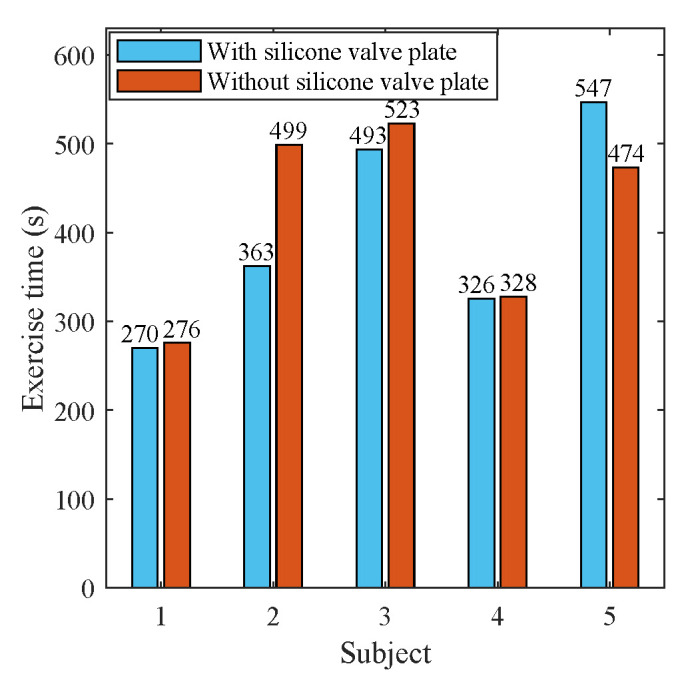
The exercise time of 10 tests in movement mode.

**Figure 14:**
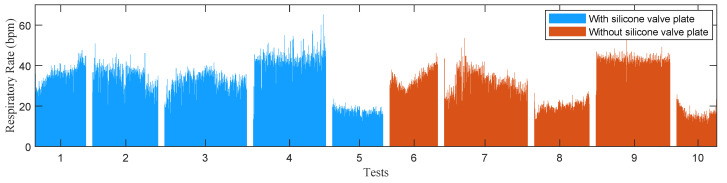
The respiratory rates of 10 tests measured by the sensor in the experiments of movement mode.

In the experiments of stationary mode, 14 subjects were instructed to wear the wireless respiration sensors and sit in a chair to breathe naturally. A button connected to the GPIO of BLE SoC was used to provide reference signals of respirations. The subjects were asked to press the button when exhaling and release it when inhaling, hence the time periods of successive button release events exactly correspond to the actual respiratory periods. The status of button, as well as the airflow temperature were sampled at a high frequency of 100 Hz and sent to a PC via an UART to USB bridge. A total of 28 tests (2592 respiratory cycles) were carried out, and the silicon valve plate of mask was removed in half of these tests (1298 respiratory cycles). In order to facilitate the comparison of different algorithms, the high frequency temperature samples saved by PC were extracted to construct samples with different sampling periods and then processed offline by different algorithms. Figure 11 and Figure 12 show the respiratory rates of 28 tests measured by the button and the recorded temperature and button status of a test. In the tests of movement mode, the sensor was evaluated by comparing the number of breaths measured by the sensor and the number of breaths counted by the subjects. The 5 subjects wearing the wireless respiration sensors were instructed to run or walk 800 meters around the standard playground. During the tests, the subjects firstly synchronized the number of breaths they counted with those measured by sensor, and then counted the number of breaths independently while running or walking. Finally, the counted number of breaths were compared with those measured by the respiration sensor to verify the effectiveness of the sensor. Each subject was tested twice, one with silicone valve plate and the other without silicone valve plate. The respiratory parameters were detected by the sensor in real-time and transmitted to the Android APP via BLE 4.0 interface for display and storage. Figure 13 and Figure 14 show the exercise time and respiratory rates of 10 tests measured by the sensor in movement mode. The sensor was also tested in high temperature environment to evaluate the effectiveness of the sensor in the aspect of respiration count and temperature difference. In the test of apnea detection, a subject was asked to simulate sleep apnea by holding his breath. In the power consumption tests, the battery of sensor was replaced with a regulated power supply of 3.7 V, and the currents were measured and logged to PC by an Agilent 34465A digital multimeter with 5000 Hz sampling frequency.

### 3.2. Results of Stationary Mode

**Figure 15:**
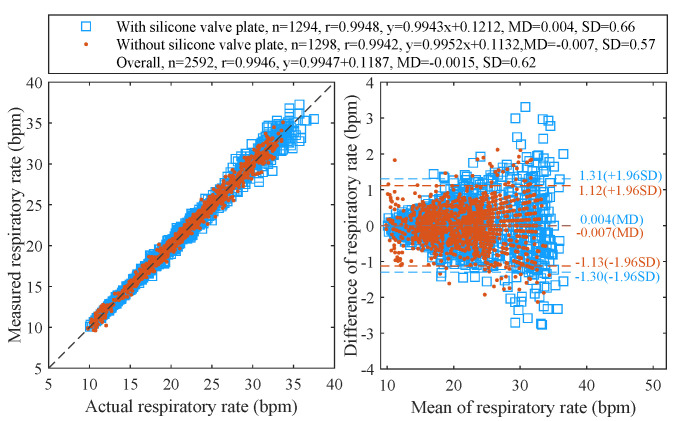
The Pearson’s correlation (left) and the Bland–Altman (right) plots of respiratory rates measured at 180 ms period.

**Figure 16:**
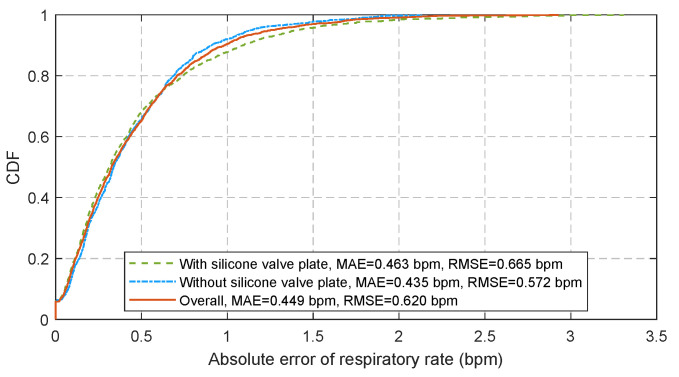
The CDF of respiratory rate estimation error at 180 ms sampling period.

**Figure 17:**
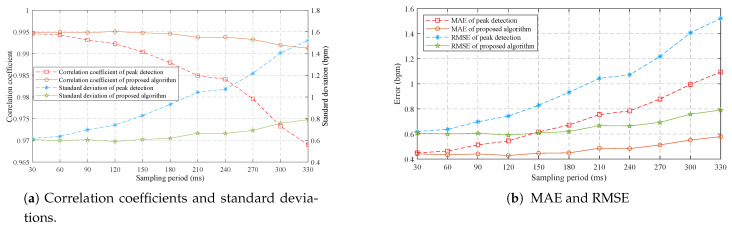
The error statistics of 28 tests (n = 2592) measured at different sampling periods.

Figure 15 displays the Pearson’s correlation and the Bland-Altman plots for 28 tests with 2592 pairs of respiratory rates measured by the proposed algorithm at a sampling period of 180 ms and the reference signals provided by the release event of button. The cumulative distribution function (CDF) of corresponding absolute errors is plotted in Figure 16. The total 2592 measurement pairs show a very high correlation coefficient (r) of 0.9946, a standard deviation (SD) of 0.62 bpm, a very small mean difference (MD) of −0.0015 bpm with 95% limits of agreement (LoA) of −1.216 to 1.213 bpm, a mean absolute error (MAE) of 0.449 bpm and a root mean square error (RMSE) of 0.620 bpm. We also compare the performance of the sensor when the silicone valve plate is installed (the blue squares in Figure 15 and the green dash line in Figure 16) and removed (the red points in Figure 15 and the blue dotted line in Figure 16). As the removal of valve plate makes the measured temperature more sensitive to respiration, the measurement pairs without valve plate demonstrate a slightly better performance than those with valve plate. Figure 17 shows the correlation coefficients, standard deviations, MAE, and RMSE of the peak detection algorithm [43] (the red dash line and the blue dash line) and our proposed algorithm (the brown solid line and the green solid line) for the 28 tests at different sampling periods. Since the timer of the BLE SoC is accurate only when the multiple of 30 ms is used as the timing unit, the step for sampling periods is set to 30 ms. As can be seen from Figure 17, the proposed algorithm can significantly reduce the negative impact of sampling period growth on the performance of correlation coefficient, standard deviation, MAE, and RMSE. As a contrast, the performance of peak detection algorithm [43] decreases seriously with the increase in sampling period. The sampling period of 180 ms is a compromise value, after which the errors increase with sampling period gradually.

### 3.3. Results of Movement Mode

**Figure 18:**
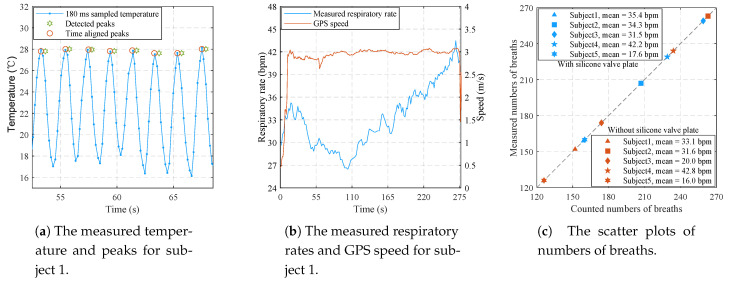
The experimental results of 10 tests in movement mode.

Figure 18a shows the partial temperature and respiratory parameters recorded by the Android APP for subject 1 in the test without silicone valve plate. The peaks of temperature signals are in good agreement with the time aligned peaks which are 360 ms ahead of the detected peaks. The delay of 360 ms is mainly caused by the spline interpolation. Figure 18b shows the measured respiratory rates and GPS speed of the complete test. The measured respiratory rates are also consistent with the self perception of subject 1. As can be seen from Figure 18c, the numbers of breaths measured by the proposed sensor are exactly the same as the numbers of breaths counted by subjects, demonstrating the effectiveness of the proposed sensor in movement mode.

### 3.4. Results in High Temperature Environment

**Figure 19:**
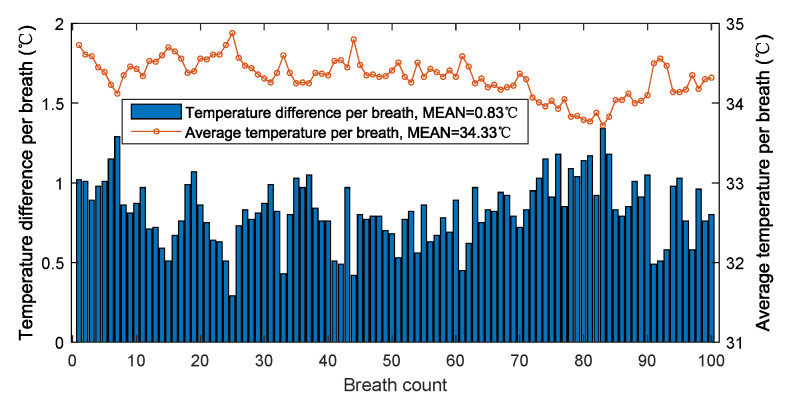
The temperature difference and average temperature per breath in high temperature environment.

Figure 19 shows the temperature difference and average temperature of 100 breaths in a hot summer day with reported ambient temperature of 34 ${}^{\circ }$C. The average temperature and the average temperature difference of 100 breaths are, respectively, 34.33 ${}^{\circ }$C and 0.83 ${}^{\circ }$C. Although the ambient temperature is very close to the high temperature alarm of 35 ${}^{\circ }$C, the temperature difference caused by breathing is still very obvious, allowing each breath to be accurately detected. Theoretically, as the ambient temperature continues to rise, the temperature difference will decrease. However, at this time, the temperature is higher than the alarm temperature, which is not suitable for people’s outdoor activities.

### 3.5. Results of Apnea Detection

**Figure 20:**
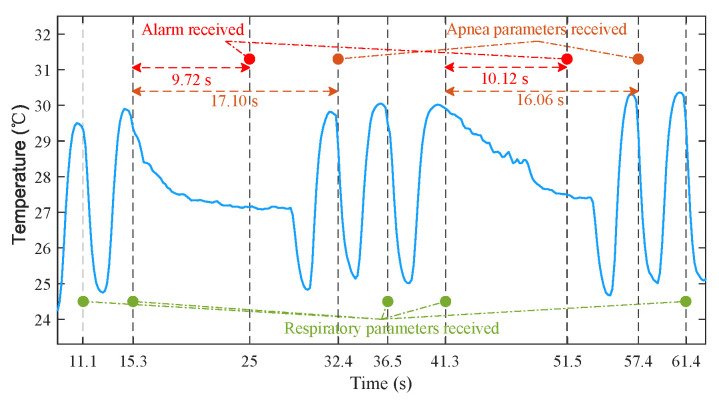
The example of apnea detection process.

The process of apnea detection is as follows. An apnea is considered to occur if no new peak is detected for 9.9 s (55 samples). Then, the sensor sends an alarm to the Android APP via BLE notification. Finally, the apnea parameters are calculated by sensor and transmitted to the APP once a new peak is detected. Figure 20 shows the detection process of the artificially simulated apnea. The data were recorded by the APP. Due to the delay of two sampling periods introduced by spline interpolation and BLE communication, the time for APP to receive the parameters of respiration and apnea was slightly behind the time of peaks. For all that, the alarm time of 9.72 s and 10.12 s was still very close to 9.9 s, and the durations of 17.13 s and 16.07 s measured by the proposed sensor were also very close to the apnea durations of 17.1 s and 16.06 s calculated from the time stamps provided by Android. The above results demonstrate that the system can identify the time lag in the temperature changes from the breathing. In future work, we will look for real patients who suffer from sleep apnea to further verify the performance of sensor.

### 3.6. Results of Power Consumption

**Figure 21:**
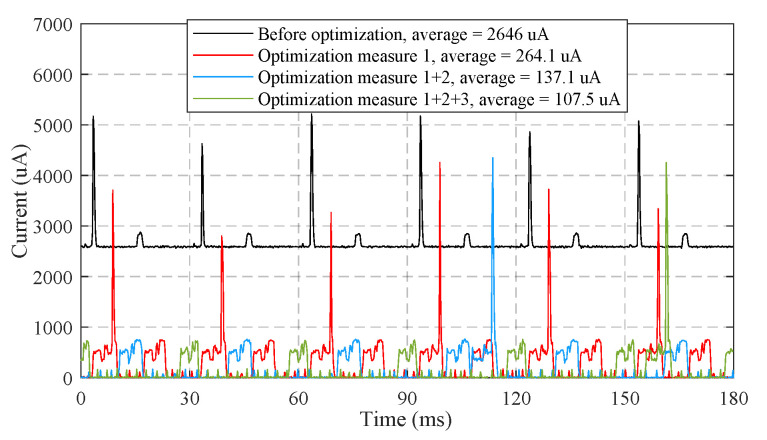
The measured currents of sensor under different optimization measures.

**Figure 22:**
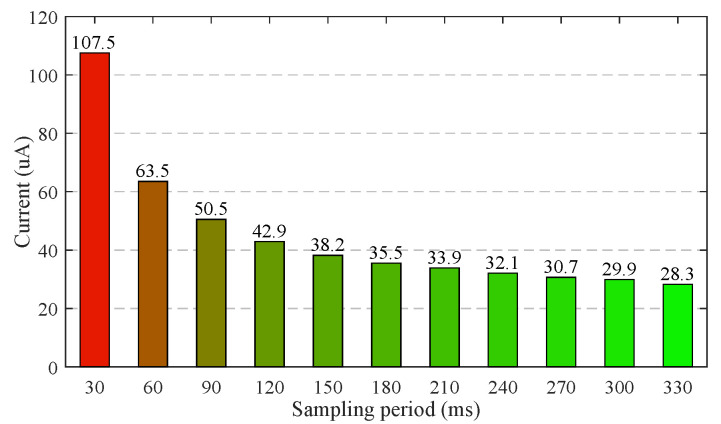
The measured currents of sensor at different sampling periods.

As demonstrated in Figure 21, the optimization measures 1–3 proposed in Section 2.6 reduce the average working current of 120 s time window from 2646 μA to 107.5 μA. Figure 22 shows the measured average working currents at different sampling periods, the working current can be further reduced by extending the sampling period. The average working current at the sampling period of 180 ms is as low as 35.5 μA. Hence, a 60 mAh Li-polymer battery is sufficient for a theoretical battery life of up to 70 days.

## 4. Discussion

Continuous, non-invasive, and accurate monitoring of respiratory rate in daily life has broad application prospects in the fields of early disease diagnosis, sleep quality analysis and exercise intensity tracking. However, there is a lack of consumer-grade respiration monitor that meets the above requirements. Addressing this issue, we provided the detailed design and evaluation of a reusable wireless wearable sensor measuring the temperature at the exhalation valve of KN95 mask. The novelties of sensor can be summarized as follows.

- The sensor detects breathing by sensing airflow temperature from vent holes of KN95 mask instead of chest or abdomen movements, hence is immune to motion artifacts and can be used in all scenarios including sleep, exercise, and other daily activities. As the vent holes of KN95 mask are the only pathway of exhaled airflows, the sensor can accommodate both breathing pathways of nose and mouth;
- We propose a lightweight signal processing pipeline to achieve high accuracy (0.449 bpm MAE) of respiratory rate measurement with very low sampling frequency (5.56 Hz) and computational complexity (an average computation time of 277.6 μs for 30 ms sampling period). Table 1 shows the comparisons between our system and the state-of-the-art systems across sensor type, accuracy performance, sampling frequency, and time window. The accuracy of our system is superior to the counterparts in smaller time window and lower sampling frequency;
- We take comprehensive measures to reduce power consumption in both hardware and software design. The sensor only consumes 131.4 μW in working mode and 55.96 μW in standby mode, hence a long endurance time can be achieved with a miniaturized battery. Table 2 shows the comparison between our system and state-of-the-art systems across power consumption. The current consumption of the proposed sensor is far less than those of other existing systems;
- We design a 3D printed cover to mount the sensor, as well as the battery, and to replace the original exhalation valve cover of the off-the-shelf KN95 mask. As the 3D printed cover can be easily fasten to and disassembled from the exhalation valve base of KN95 mask without any assembly unit, the sensor can be reused conveniently. The sensor can also be adapted to other off-the-shelf KN95 masks by redesigning the matched covers. In addition, the installed sensor only adds a very light weight of 8.94 grams to the KN95 mask, imposing little impact on wearing comfort;
- The sensor has a very simple circuit structure and is only made up of off-the-shelf electronic components. The bill of material (BoM) cost is as low as USD 3.67.

Due to budget constraints and epidemic prevention policy, we have not compared our system with polysomnography (PSG), which is a primary clinical tool for respiratory monitoring. Instead, we use the button status provided by the subjects as the reference signal. The button was pressed on when exhaling and released off when inhaling. From the analysis of respiratory mechanism, the flow time of airflow in the trachea will cause the peak and valley of temperature to lag behind the real inspiratory start time and expiratory start time. Due to the subject’s response time, the start time of inhalation and exhalation measured by button will also introduce lags. Fortunately, since the respiratory period is obtained by subtracting the time of two consecutive peaks, the above lag will be eliminated by subtraction. Figure 12 shows the recorded temperature and button status in a test of stationary mode. The lags between the detected peaks of temperature and the release events (transition from on to off) of the button, as well as the lags between the detected troughs and the press events (transition from off to on) are relatively consistent. Therefore, these delays will not affect the measurement of respiratory period. In future work, we will compare the peaks and troughs of temperature signal with the onset time of inspiration and expiration provided by professional medical equipment, and further investigate the influence of lags.

Breathing patterns vary from person to person. The bandwidth of fast breathing is greater than that of slow breathing. Hence, the temperature sensor should provide with high bandwidth (fast response time or small time constant) to capture the temperature variations induced by respiration. When the bandwidth of the temperature signal exceeds the bandwidth of the sensor, the measured temperature amplitude will be attenuated. We select the Murata NCP15WL473J03RC NTC sensor with a small time constant to avoid the amplitude attenuation. In the experiments of stationary and movement modes, the sensor has demonstrated its effectiveness for respiratory rates from 9.8–65.2 bpm shown in Figure 11 and Figure 14. This range basically covers the respiratory rates of bradypnoea (<12 bpm), normal (12–20 bpm) and tachypnea (>24 bpm). The temperature amplitude of each respiratory cycle is also a factor affecting whether respiration can be detected. The small amplitude appears in light breath or high temperature environment. We use the sensor with small time constant to minimize the amplitude attenuation, and the voltage divider with controllable resistors to improve the measurement gain. In the experiment of high temperature environment, the sensor still works at the average temperature of 34.43 ${}^{\circ }$C and the average amplitude of 0.83 ${}^{\circ }$C. Theoretically, as the amplitude continues to decrease, breathing will begin to be undetectable. In future work, we will further explore the limits of sensors in this regard.

Detection threshold is also an important parameter to be carefully tuned. To accommodate different respiration intensity, we have tried to dynamically adjust the detection threshold according to the temperature difference of previous respiratory cycles. However, in the experiments, we found that respiration may go undetected when the detection threshold remains high due to previous respiratory cycles and the temperature difference suddenly decreases. Therefore, in practical applications, the detection threshold is still empirically set to 0.2 ${}^{\circ }$C for sleep monitoring and 0.4 ${}^{\circ }$C for exercise monitoring.

The proposed wireless wearable sensor detects respiration by sensing airflow temperature from the exhalation valve of a KN95 mask. For masks without exhalation valve, we have not test them with the proposed sensor. Since the exhaled airflow of mask without exhalation goes out through the multilayer composite fabrics, the temperature difference caused by breathing will be smaller than that of the mask with exhalation valve, making it difficult to detect breathing. To solve this problem, we will explore the best sensor installation position for masks without an exhalation valve in future work. In addition, textile sensor will also be installed on the mask without exhalation valve to detecting respiration by sensing the strain variation of multilayer composite fabrics.

As an accurate, low cost, and easy-to-use solution for respiration monitoring, the wireless wearable sensor will have a very broad application prospect in the fields of early diagnosis of diseases, such as COVID-19, cardiopulmonary arrest, obstructive sleep apnea, and so on, classification of sleep stages, and exercise monitoring.

## 5. Conclusions

In this paper, a wireless wearable sensor for non-invasive, real-time, and accurate respiration monitoring using an off-the-shelf KN95 mask has been designed, implemented, and evaluated. By sensing the airflow temperature at the exhalation valve of the KN95 mask, the sensor can accurately measure the parameters of every respiration and apnea, regardless of the breathing pathways through nose or mouth, and the application scenarios of sleep or exercise. We design a 3D printed cover to install the sensor hardware and to replace the original cover of the KN95 mask, making the sensor to be easily reused. We also propose a light weight signal processing pipeline running at a very low sampling frequency to detect respiration and apnea, and low power optimization measures to extend battery life. The main advantages of the designed sensor are its high accuracy, low cost, long battery life, miniaturization, and convenience. In the comprehensive experiments, the designed sensor shows a small MAE of 0.449 bpm and a very low power consumption of 131.4 μW, demonstrating its promise in practical applications. Our future work will focus on the sleep stage detection based on these respiratory data and AI technology. 

## Figures and Tables

**Table 1:** Performance compared to other sensors.

System	Sensor Type	Accuracy Performance	Sampling Frequency	Time Window
This paper	NTC series resistors	LoA = –1.216/0.647/0.173∼1.213/0.638/0.165 bpm MAE = 0.449/0.238/0.063 bpm RMSE = 0.620/0.328/0.086 bpm Accuracy = 98.01/98.94/99.72%	5.56 Hz 180 ms	1/2/8 cycles (2.95/5.9/23.6 s)
[9]	Bio-impedance	LoA = –4.97∼3.63 bpm	256 Hz	8 cycles
[12]	ECG	MAE = 0.3/0.2 bpm	300 Hz	32/64 s
[13]	PPG	RMSE = 0.48549 bpm	125 Hz	30 s
[18]	piezoresistive	MAE = 0.45 bpm (static) MAE < 1.86 bpm (walking)	Unknown	20 s
[22]	FIR+NIR cameras	LoA = –2.51∼3.46 bpm	8.7 Hz	12 s
[23]	Depth images	LoA = –1.9∼2.3 bpm	60 Hz	2 min
[24]	RFID	Accuracy = 98%	64 Hz	25 s
[25]	900 MHz RF signals	MAE = 0.13 bpm	Unknown	50 s
[26]	WiFi CSI	Accuracy = 98%	20 Hz	Unknown
[27]	Doppler radar	MAE = 0.38 bpm (average of 6 signals)	50 Hz	8 s
[29]	FMCW radar	MAE < 0.747 bpm	384.6 Hz/2.6 ms	20 s
[31]	Inertial sensors	MAE = 1.8 bpm (23 cycles)	32Hz	60 s
[32]	Magnetometer	MAE = 0.5 bpm	10 Hz	1 cycle

**Table 2:** Power consumption compared to other sensors.

System	Sensor Type	Power Consumption
Our	NTC series resistors	35.5 μA/131.4 μW
[9]	Bio-impedance electrodes	∼5.8 mA
[20]	Inductive strain sensor	<7 mA/23.1 mW
[28]	24 GHz medical radar	1000–1500 mA
[31]	Inertial sensors	12 mA
[32]	Magnetometer	<1 mA
[44]	Hot-film flow sensor	43 mW
[45]	Humidity sensor	7–25 mA

## Data Availability

The data presented in this study are available on request from the corresponding author.
